# Fast digital lossy compression for X-ray ptychographic data

**DOI:** 10.1107/S1600577520013326

**Published:** 2021-01-01

**Authors:** Panpan Huang, Ming Du, Mike Hammer, Antonino Miceli, Chris Jacobsen

**Affiliations:** a Department of Physics and Astronomy, Northwestern University, Evanston, IL 60208, USA; b Advanced Photon Source, Argonne National Laboratory, Argonne, IL 60439, USA; c Chemistry of Life Processes Institute, Northwestern University, Evanston, IL 60208, USA

**Keywords:** X-ray ptychography, pixel array detectors, lossy compression

## Abstract

Increases in X-ray brightness from synchrotron light sources lead to a requirement for higher frame rates from hybrid pixel array detectors (HPADs), while also favoring charge integration over photon counting. However, transfer of the full uncompressed data will begin to constrain detector design, as well as limit the achievable continuous frame rate. Here a data compression scheme that is easy to implement in a HPAD’s application-specific integrated circuit (ASIC) is described, and how different degrees of compression affect image quality in ptychography, a commonly employed coherent imaging method, is examined. Using adaptive encoding quantization, it is shown in simulations that one can digitize signals up to 16383 photons per pixel (corresponding to 14 bits of information) using only 8 or 9 bits for data transfer, with negligible effect on the reconstructed image.

## Abstract 2

A lossy adaptive encoding scheme that can be implemented on a per-pixel basis in hybrid pixel array detectors is described. It is shown that this lossy compression scheme has no effect on X-ray ptychographic images even at low signal levels.

## 1. Introduction   

The brightness of synchrotron light sources for X-ray experiments has been increasing dramatically over the past decades, with diffraction-limited storage rings beginning to deliver the next advance (Eriksson *et al.*, 2014 ▸). Many light source experiments require hybrid pixel array detectors (HPADs), where a semiconductor sensor is paired with an application-specific integrated circuit (ASIC) to record a two-dimensional intensity distribution. These HPADs can be used for direct image recording when sufficient geometric image magnification is used (Vagovič *et al.*, 2013 ▸; Blackhall *et al.*, 2014 ▸), but they are more commonly used to record far-field diffraction intensities in crystallography, in photon correlation spectroscopy, and in coherent diffraction imaging methods such as ptychography.

When an X-ray photon of energy *E* is absorbed in an HPAD’s sensor, an electron–hole separation charge 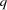 = 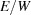 is generated, where 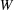 = 3.65 eV in the case of a silicon sensor (Fraser *et al.*, 1994 ▸). In a photon-counting detector, this charge is collected and a photon is counted by the ASIC when this charge exceeds a threshold value which is some fraction of *q*. However, it takes a finite time to collect this charge due to the transport properties of the sensor, leading to a ‘dead-time’ 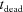 before another photon can be successfully detected. For this reason, photon-detecting HPADs usually have a per-pixel count rate limit of about 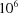
 photons s^−1^ (Trueb *et al.*, 2012 ▸). This is begining to limit their application with increasingly bright X-ray sources, where many photons arrive within specific time intervals due to the electron bunch structure in the storage ring. In contrast, charge-integrating detectors become favored as X-ray brightness increases because they do not have an intrinsic limit to photon arrival rate, even in the case of X-ray free-electron lasers (XFELs) where all the photons might arrive within 20 fs. In these detectors, a total charge 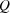 = 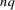 is collected from *n* photons during an acquisition time 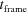, leading to a voltage 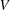 = 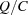 over a collection capacitance *C*. This voltage then leads to an analog detection unit (ADU) of ADU = *aV*, where *a* indicates the calibration of an analog-to-digital converter (ADC). Thus, in the end, the digitized signal per acquisition time 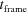 is given by
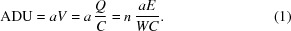
In the following, we will assume that 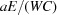 = 1 for simplification.

In a charge-integrating HPAD, one must periodically integrate the collected charge *Q* and either store that information on the ASIC or transfer it off the detector immediately (Graafsma *et al.*, 2016 ▸). Some HPADs developed for XFEL applications include per-pixel capacitors on the ASIC to store the charge *Q* for up to eight frames at a burst frame rate up to 10 MHz (Philipp *et al.*, 2016 ▸), or 352 frames at a burst frame rate of 6.5 MHz (Henrich *et al.*, 2011 ▸), followed by digitization of the charge on each capacitor and subsequent digital transfer of the detected frames. However, the maximum *continuous* frame rate in these detectors is no higher than 16 kHz (Allahgholi *et al.*, 2019 ▸), or 20 kHz as anticipated by near-term extension of the ePix detectors (Blaj *et al.*, 2016 ▸). The common bottleneck limiting frame rate in charge-integrating HPADs is data bandwidth (Graafsma *et al.*, 2016 ▸), and it can limit bandwidth in photon-counting detectors as well. Some detectors such as the EIGER can be configured to store data in different bit depths (Dinapoli *et al.*, 2011 ▸). At low incident fluence into all detector pixels, one can switch to a mode with a lower bit depth to increase the frame rate. However, this does not solve the problem at high fluence, or for situations where some pixels (for example, near the center of coherent diffraction patterns) see high fluence while others do not. Another way to reduce data bandwidth requirements is to design the ASIC with per-pixel analog-to-digital conversion followed by lossy compression. Immediate digitization also has the advantage of reducing signal distortion, analog noise addition, and interference between pixels. However, one must then consider how lossy compression affects the information obtained from an experiment. This is what we consider for the case of X-ray ptychographic imaging.

## 2. X-ray ptychography   

Ptychography is an imaging method where a spatially limited coherent beam (the probe) illuminates a series of overlapping positions on an extended specimen, with far-field diffraction patterns recorded at each probe position (Hoppe, 1969*a* ▸,*b* ▸). An iterative algorithm (Faulkner & Rodenburg, 2004 ▸) is then used to recover the phase in the set of diffraction patterns, and reconstruct the magnitude and phase of the exit wave leaving the specimen with a spatial resolution limited not by the size of the probe beam but by the largest diffraction angle at which significant scattering is recorded and phased. Following its first demonstration in X-ray imaging (Rodenburg *et al.*, 2007 ▸), X-ray ptychography has been adopted widely, achieving sub-10 nm spatial resolution (Shapiro *et al.*, 2014 ▸) and being used for 3D imaging via ptychographic tomography (Dierolf *et al.*, 2010*a* ▸). Reconstruction algorithms have been extended (Thibault & Menzel, 2013 ▸) to allow the probe to be scanned rapidly across the specimen (Pelz *et al.*, 2014 ▸). However, the frame rate of currently available X-ray HPADs sets a limit to high-throughput imaging demonstrations (Deng *et al.*, 2019 ▸), so that further advances in throughput will require frame rates well above what is currently available (Jacobsen *et al.*, 2017 ▸).

It is desirable to limit the photon fluence (cumulative number of photons incident per area on the specimen) both to speed up imaging time and also to minimize the X-ray radiation dose deposited in the specimen. At expected signals of *N* photons per detector pixel, one will have fluctuations between different measurements of the same intensity due to Poisson statistics with a standard deviation of 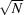. The signal-to-noise ratio is then expected to be proportional to 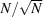 = 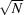. A number of studies have addressed the performance of iterative phase retrieval algorithms at these low photon exposures (Huang *et al.*, 2009 ▸; Schropp & Schroer, 2010 ▸; Godard *et al.*, 2012 ▸; Jahn *et al.*, 2017 ▸; Hagemann & Salditt, 2017 ▸; Du *et al.*, 2020 ▸). These studies have generally shown that the achievable spatial resolution is consistent with what one would expect based on the fluence, and the specimen’s intrinsic contrast. In addition, no scattering-angle-limiting and inefficient optics are placed between the specimen and the detector in ptychography, again minimizing the radiation dose associated with imaging at a given spatial resolution and specimen contrast.

## 3. Ptychography with lossy compressed data   

Our goal is to understand how varying degrees of lossy data compression affect the quality of ptychographic image reconstructions. While our work is motivated by an interest in incorporating lossy data compression on the ASIC of an HPAD, lossy data compression has been demonstrated in X-ray ptychography for two different purposes: decreasing the disk storage space of a dataset (Loetgering *et al.*, 2017 ▸), and increasing the number of diffraction patterns (detector frames) that can be processed within the memory limit of a graphical processing unit (GPU) (Wakonig *et al.*, 2020 ▸). In the first example of decreasing disk storage space, two methods were tested: the use of singular value decomposition compression (SVDC) on the set of obtained diffraction patterns, and storing sums from non-contiguous pixels in an approach called constrained pixel sum compression (CPSC). These approaches showed a slight loss of spatial resolution when lossy SVDC was used, and a larger decrease in spatial resolution when CPSC was used (Loetgering *et al.*, 2017 ▸). In the second case, the software package *PtychoShelves* (Wakonig *et al.*, 2020 ▸) uses a scheme where the actual value 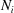 for the signal at pixel *i* is encoded via a quantization step (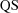) into a compressed value 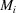 of
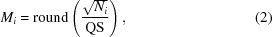
so as to use the Gaussian scaling that approximates Poisson statistics. With 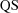 = 0.5, this means that 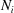 = 16320 is stored as 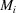 = 255, compressing nearly 14 bits of raw signal dynamic range into an 8 bit integer. One can then go from the encoded value 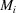 back to a decoded value 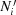 using
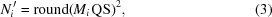
which does not exactly reproduce the results of Fig. 10 of Wakonig *et al.* (2020 ▸), though the results of that figure and of equation (3) are both within 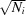 of the correct value 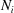 so that they both reproduce the signal within one standard deviation of the Poisson distribution. To test the effects of lossy compression, they simulated the generation of a ptychographic dataset with Poisson-distributed signal-dependent noise, compressed it with varying values of 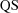, and evaluated the resulting reconstructed image using two metrics. The first was the signal-to-noise ratio calculated using the ground truth object; this gave a signal-to-noise ratio that stayed within 5% of the uncompressed value with 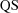 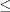 0.5, and within 20% of the uncompressed value with 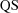 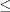 1.0. The second was the achieved spatial resolution of the reconstructed image. They calculated the Fourier ring correlation (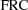) as a function of scattering angle to judge the spatial resolution based on a half-bit 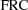 threshold criterion (van Heel & Schatz, 2005 ▸). They found essentially no change in the spatial resolution of the reconstructed image with values of 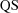 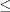 1.9.

We wished to consider lossy compression schemes that can be implemented on a per-pixel basis. By doing so, one reduces the volume of data that must be channeled per pixel to the data output region of the ASIC in an HPAD, both increasing the aggregate data bandwidth of the ASIC and also reducing wiring demands on the ASIC layout. Even if one uses further compression downstream of the individual pixel level within the ASIC, or off-ASIC compression such as in a field-programmable gate array (FPGA), compression at the origin of per-pixel data will increase the throughput of such subsequent data handling elements (Hammer *et al.*, 2020 ▸). While the lossy compression scheme of equation (2) can be written with a simple mathematical formula, it is less straightforward to implement on a per-pixel basis in an ASIC due to the use of square root and division operations. [One example (Suresh *et al.*, 2013 ▸) of an on-chip implementation of a floating point square root calculation involves an area of 118 µm^2^ in a 65 nm node process, which is comparable with the entire area of one pixel in many HPADs and thus clearly impractical to implement on a per-pixel basis.] The method of lossy SVDC is even more difficult to implement on the ASIC, in part because it requires access to the entire dataset before compression can take place. The constrained pixel sum compression (CPSC) method is more amenable to implementation on a HPAD ASIC, but it showed a more significant degradation in spatial resolution.

**Figure 1:**
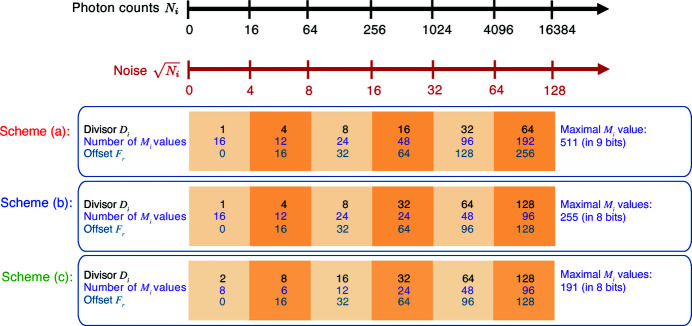
Three different lossy compression schemes (*a*), (*b*), and (*c*) which all use the approach of equation (4) though with different values of 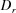 and 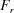. Scheme (*a*) is also detailed in Table 1 ▸.

We therefore consider an alternative lossy compression scheme that recognizes the intrinsic signal-dependent Poisson error as equation (2) does, but which is easier to incorporate in simple per-pixel ASIC circuitry. It involves three steps that are easy to carry out in integer mathematics: (i) comparison with a 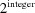 boundary value to determine an integer range value *r*, (ii) division by a 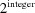 number 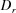 which is a bitwise shift unique to each count region as shown in Fig. 1 ▸, and (iii) an addition of a 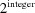 offset number 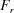. The formula for lossy encoding an actual count 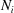 to an encoded value 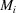 is
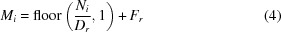
where the 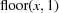 function truncates any non-integer result of the division of *x*, as is the case with division implemented as a bitwise shift. The formula for decoding 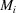 to a lossy recovered value 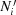 is
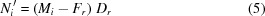
where multiplication by 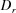 can again be done using a bitwise shift (though since this decoding will be done on the computer that processes the data, rather than on the detector ASIC that encodes the data, this multiplication can alternatively be done as a floating point operation). This encoding logic is expected to occupy a 25 µm × 25 µm space for each pixel with pixel size of around 100 µm in 65 nm technology (Hammer *et al.*, 2020 ▸). An example of an implementation of this approach is shown in Table 1 ▸, where the values of 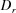 and 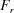 used are those of scheme (*a*) shown in Fig. 1 ▸. As can be seen, this produces gaps in the sequence of 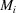, but it allows a 14 bit integer (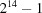 = 16383) to be stored within a 9 bit integer (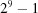 = 511) with an error that never exceeds one standard deviation.

**Figure 2:**
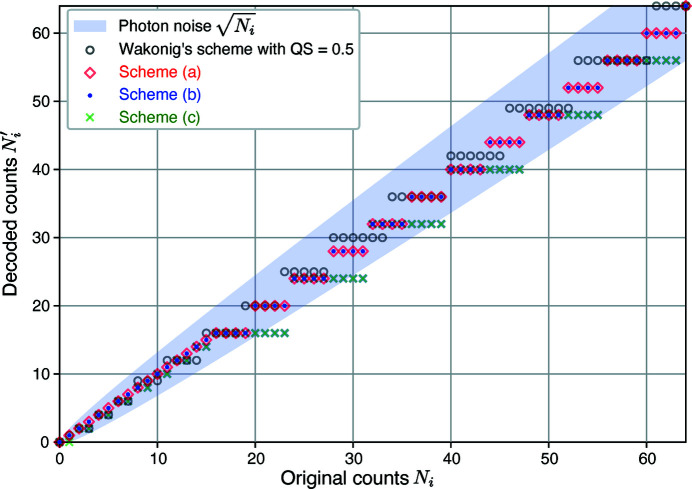
Decoded photon count values 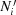 as a function of original photon counts 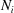 for various lossy encoding schemes. Schemes (*a*), (*b*), and (*c*) are the main methods we test below, with all three schemes using equations (4) and (5) with values of 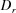 as shown in Table 1 ▸. We also show a lossy encoding scheme used by Wakonig *et al.* (2020 ▸) which uses equations (2) and (3) with 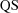 = 0.5, though the Wakonig scheme is not easily implemented in per-pixel ASIC circuitry due to its use of non-integer square root and division operations. All methods except for scheme (*c*) produce decoded values 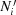 that are within one standard deviation 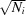 of the original values 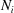 (indicated by the blue shaded area) for 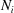 up to 64 counts, using the Poisson distribution with a mean equal to each value of 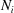.

In order to test the effects of increasing degrees of lossy compression in ptychographic image reconstruction, we show in Fig. 1 ▸ the above scheme (*a)*, but also schemes (*b*) and (*c*) which provide increasing degrees of lossy data compression through larger values of 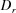. We also show in Fig. 2 ▸ the decoded values 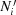 for photon numbers 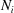 for our lossy compression schemes (*a*), (*b*), and (*c*), and also for the scheme of equations (2) and (3) with 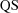 = 0.5. As can be seen, for the original counts up to 64 counts, all the encoding methods except for scheme (*c*) deliver decoded values 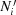 that are within 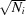 of 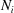 (that is, within one standard deviation of the original value 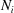). With scheme (*c*), some decoded values 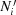 are slightly more than one standard deviation away from the original value 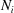.

## 4. Effect of lossy compression on ptychographic imaging   

**Figure 3:**
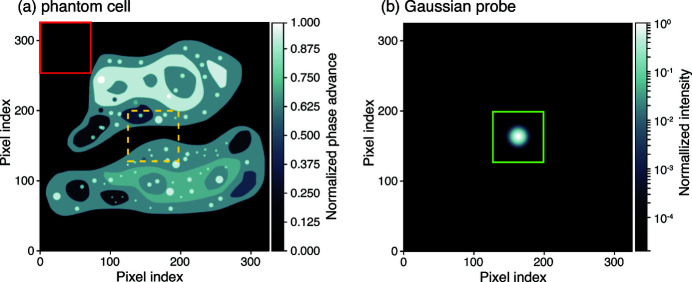
Phantom cell object (*a*) and limited-area coherent illumination spot [(*b*), the ‘probe function’] used for our simulations. The 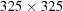 pixel phantom cell is the same pure-phase object used in previous studies of the effect of changing photon fluence in different X-ray microscopy methods (Hagemann & Salditt, 2017 ▸; Du *et al.*, 2020 ▸). The probe function’s intensity is shown at the same scale here, but it is in fact a 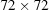 pixel array which is scanned across the phantom, with the boundaries of the probe array indicated by a green box. Within-‘cell’ and out-of-‘cell’ regions used in Fig. 4 ▸ are indicated by the yellow and red boxes, respectively.

As noted in Section 3 above, the effects of the lossy ptychographic data compression scheme of equations (2) and (3) on ptychographic image quality have already been studied (Wakonig *et al.*, 2020 ▸), showing that values of 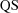 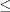 0.5 produce almost no observable difference in image correlation, or in spatial resolution as measured by the 1/2 bit threshold of the 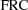. Here we wish to carry out a similar test of the effects of the lossy compression method of equations (4) and (5) with parameters for schemes (*a*), (*b*), and (*c*) as indicated by Fig. 1 ▸. In order to do this, we will use as the true object a two-dimensional phantom that has been designed to resemble a biological cell, and a Gaussian illumination spot (which approximates the focal spot produced by various types of X-ray nanofocusing optics) as shown in Fig. 3 ▸. This phantom was developed as part of a comparison of the fluence requirements of in-line Fresnel holography versus far-field coherent diffraction imaging (Hagemann & Salditt, 2017 ▸), and the same phantom has been used in a more recent comparison of the fluence requirements of both far-field and near-field ptychography along with in-line Fresnel holography (Du *et al.*, 2020 ▸). Because X-ray phase is advanced rather than retarded in materials (Larsson *et al.*, 1924 ▸; Jacobsen, 2020 ▸), the original phantom (Hagemann & Salditt, 2017 ▸) was modified by taking its complex conjugate (Du *et al.*, 2020 ▸). Within the 48.2% of the pixels that comprise the phantom ‘cell’ in the entire array, the optical modulation on the incident illumination imparts a mean phase of 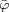 = 0.643 rad, a single-pixel variance of 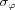 = 0.037 rad, and a bound of 0 to 1 rad (this object phase contrast is representative of what one might have in soft X-ray imaging; the contrast is usually lower in hard X-ray imaging). Object variations with 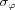 = 0.037 happen over length scales of a single pixel, while photon statistics also produce variations at the single pixel level. Therefore one can ask that the object’s phase variations become greater than or equal to noise fluctuations by requiring
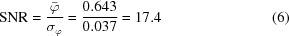
in this case. Prior studies (Du *et al.*, 2020 ▸) have shown that one can then estimate the required fluence from
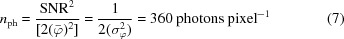
for this phantom’s parameters. This is in fact observed in 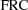 crossing curves in simulation studies (using the exact same phantom) of near-field holography [Fig. 4 of Hagemann & Salditt (2017 ▸]), and both near-field holography and far-field ptychography [Fig. 6 of Du *et al.* (2020 ▸)].

**Figure 4:**
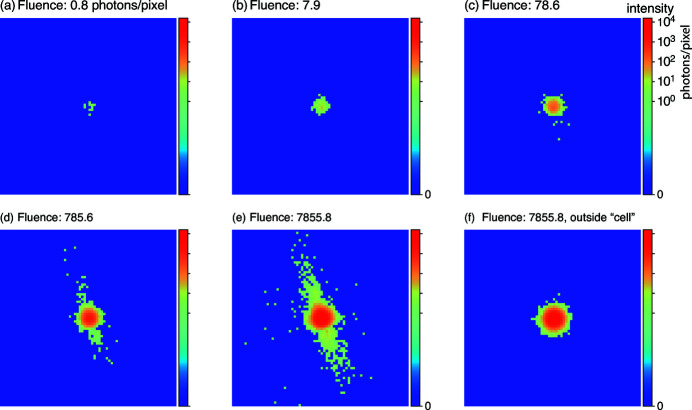
Example diffraction patterns calculated for a single exposure of the phantom cell of Fig. 3 ▸(*a*), with Poisson noise corresponding to an integrated fluence 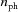 (photons per pixel) from the set of all probe positions that illuminate a single pixel of the phantom. The same intensity scale in photons per pixel in the diffraction pattern is used in all cases. The single diffraction patterns (*a*)–(*e*) are for the probe centered on the position of the yellow box in Fig. 3 ▸(*a*), while diffraction pattern (*f*) is for the probe centered outside the phantom ‘cell’ at the position of the red box in Fig. 3 ▸(*a*). This figure shows how little signal is recorded at high spatial frequencies (large radii from the diffraction pattern center) from phantom cell features, so that the preservation of low level signals in our lossy encoding schemes is especially important.

In order to understand the effects of lossy compression in far-field ptychography at various fluences 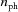, we followed the same approach used in a previous comparison of the fluence dependence of a variety of coherent X-ray imaging methods (Du *et al.*, 2020 ▸). We assumed a finite coherent illumination spot (the probe function) with a Gaussian distribution in both magnitude and phase, using a standard deviation of 6 pixels (FWHM ≃ 14 pixels) and a phase that varied from 0 to 0.5 rad. While many researchers use spiral probe scans to avoid grid artifacts (Thibault *et al.*, 2009 ▸; Dierolf *et al.*, 2010*b* ▸), we have modeled the use of rectangular scans which are better suited to constant-velocity continuous scanning (Pelz *et al.*, 2014 ▸; Deng *et al.*, 2015 ▸; Huang *et al.*, 2015 ▸), and which do not display artifacts if sufficient probe overlap is used (Bunk *et al.*, 2008 ▸; Huang *et al.*, 2014 ▸). The shift between probe positions was set to 5 pixels to provide a very high degree of overlap, which is of particular importance at low fluence levels (Du *et al.*, 2020 ▸). (Even so, a slight grid artifact is present which affects some 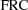 values as noted in Fig. 7.) This led to a square scan grid with 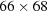 probe positions, with the probe embedded in a 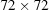 pixel array. Therefore at each probe position, the appropriate 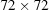 pixel subregion of the phantom was extracted as shown in Fig. 3 ▸, the phase modulation of the phantom subregion was applied, and a Fourier transform was taken to calculate the far-field diffraction intensity. We then used a random number generator with Poisson statistics to introduce noise into the diffraction pattern recorded at each probe position, with this noise based on the integrated fluence (incident photons per pixel in sample space instead of detector space) from all probe positions touching upon a single pixel. We could then choose to apply one of our encoding schemes to the set of diffraction patterns, and thus evaluate ptychographic imaging as a function of fluence for data without loss, or with our lossy compression schemes (*a*), (*b*), or (*c*). We show in Fig. 4 ▸ a set of diffraction patterns created in this way, over a wide range of fluences, and with no encoding versus lossy compression scheme (*a*).

Having obtained sets of simulated ptychographic scan data, we then carried out ptychographic image reconstruction using an approach based on automatic differentiation to calculate the gradient of the loss function using *Autograd*. *Autograd* is a Python package which differentiates standard Python, Numpy and Scipy code (Maclaurin *et al.*, 2015 ▸). The same automatic differentiation approach was used in our previous publication (Du *et al.*, 2020 ▸), which instead used *TensorFlow* as the automatic differentiation engine, but employed the same forward model and optimization algorithm.

In brief, we used a least squares (LSQ) cost function to measure the difference between the present guess 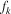 of the detected intensities based on a guess of the object, versus the ‘measured’ intensities 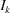 of the diffraction patterns at each probe position *k*. Automatic differentiation was then used to guide the adjustment of the object so as to minimize the difference 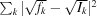.

The LSQ cost function is frequently used for the optimization strategy. In fact, the commonly used algorithm ePIE (Maiden & Rodenburg, 2009 ▸) is a special form of maximum likelihood algorithms using either LSQ or Poisson cost functions (Godard *et al.*, 2012 ▸). While reconstruction with a Poisson cost function can give sharper edge boundaries, it can also introduce fringe-like artifacts around the edges of sharp features, and the formation of fringes is sensitive to initial guess. Using a Poisson cost function also has less deterministic converging behavior than using a LSQ cost function (Du *et al.*, 2020 ▸). Moreover, the LSQ cost function was shown to have better numerical robustness in the standard deviation of the estimation (Godard *et al.*, 2012 ▸). Therefore, for our purposes of testing the detrimental effect of lossy compression on recorded ptychographic intensities, we used the LSQ noise model for all reconstructions.

## 5. Numerical experiments   

**Figure 5:**
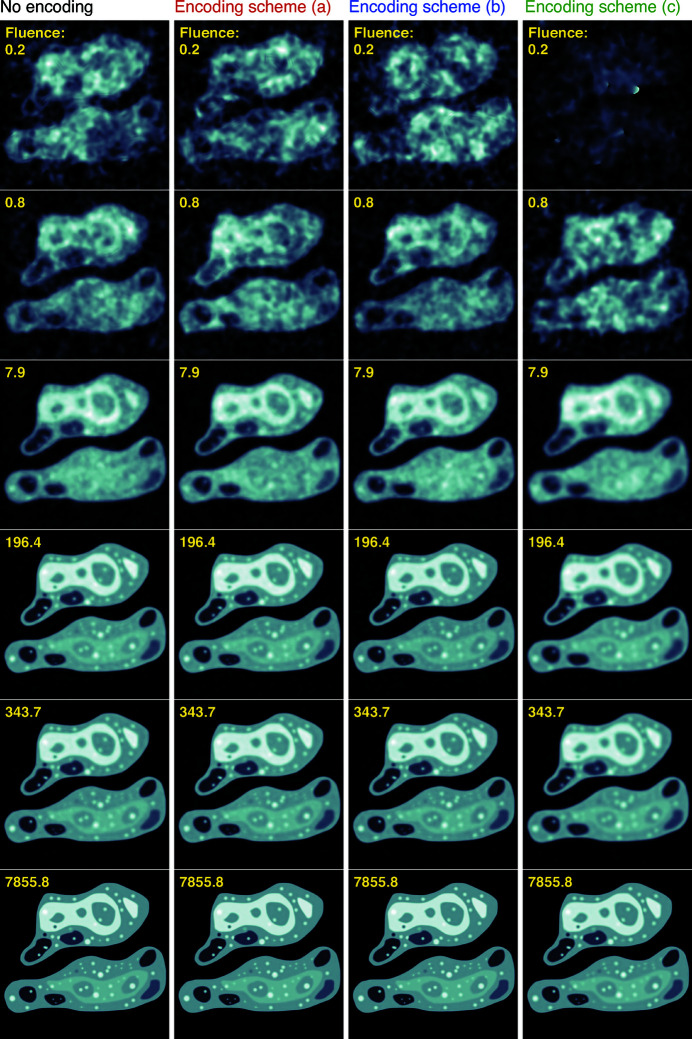
Reconstructed ptychographic images of the phantom cell of Fig. 3 ▸ obtained at photon fluences 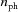 from 0.8 to 7855.8 photons pixel^−1^. The images in successive columns are from a simulated dataset with no encoding (left), and then with loss encoding schemes (*a*), (*b*), and (*c*) in the subsequent columns. These images were obtained using a least-squares cost function, rather than a Poisson noise cost function, which leads to a smoothed appearance at low fluences (Du *et al.*, 2020 ▸). Use of the lossy encoding scheme (*a*) has almost no effect on reconstructed image quality, while the increasing degree of loss provided in schemes (*b*) and especially (*c*) lead to lower quality reconstructed images at very low fluence.

**Figure 6:**
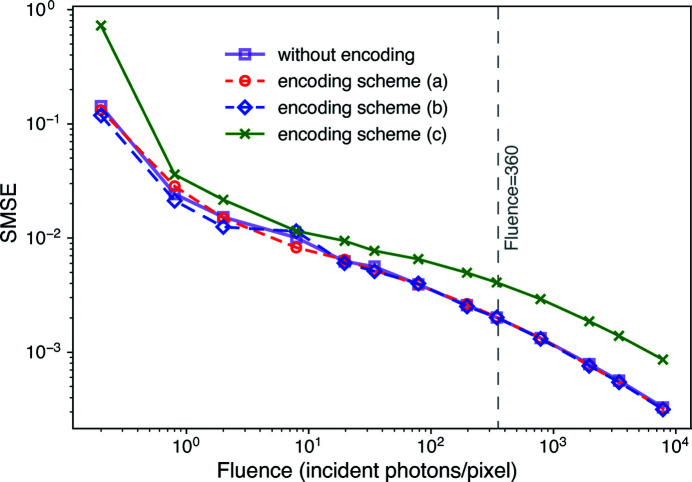
Error in the reconstructed ptychographic image as a function of fluence, for both the original ptychographic dataset and for data with the lossy encoding schemes (*a*), (*b*), and (*c*) applied. Shown here is the within-support mean-squared error (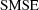) calculated using equation (8), where the support *S* corresponds to the set of pixels in the phantom (Fig. 3 ▸) that contain ‘cell’-like features. As can be seen, it is only when one uses the more aggressive lossy encoding scheme (*c*) that one obtains a noticable increase in the 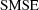 error, and this happens at all fluences. Also indicated is the estimate of equation (7) for the critical fluence of 360 photons pixel^−1^.

Using the simulated data sets and reconstruction method outlined above, we obtained reconstructed ptychographic images over a wide range of fluences 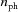 ranging from 0.8 to 7855.8 photons per pixel assuming a beam energy of 5 keV, and with no encoding or the lossy encoding schemes (*a*), (*b*), and (*c*). (At the very lowest fluences, the maximum signal in any given pixel is quite low so one could use fixed 8 bit data depth with no need for compression, but we wish to demonstrate a lossy compression method that will work at the higher fluence levels required for high fidelity imaging of low contrast features.) As can be seen in Fig. 5 ▸, the use of lossy encoding scheme (*a*) (described in Table 1 ▸ and Fig. 1 ▸) leads to little or no reduction in image quality, even at very low fluences. However, by the time one reaches the more aggressive compression found in lossy scheme (*c*), there is a noticeable affect on image quality. In order to quantify this, we used two metrics. The first involves defining a finite support region *S* within all pixels *j* that contains the cell-like features in the phantom. We then calculated the within-support mean squared error (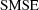) of the reconstructed phase of the object using
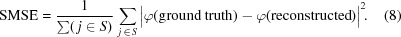
The resulting 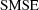 values for the images obtained using the non-encoded ptychographic data set, as well as the lossy encoded data, are shown in Fig. 6 ▸. As can be seen, there is very little change in the 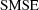 versus fluence when using lossy encoding schemes (*a*) and (*b*), while the more aggressive lossy encoding scheme (*c*) shows a significant increase in the error at all fluence values.

**Figure 7:**
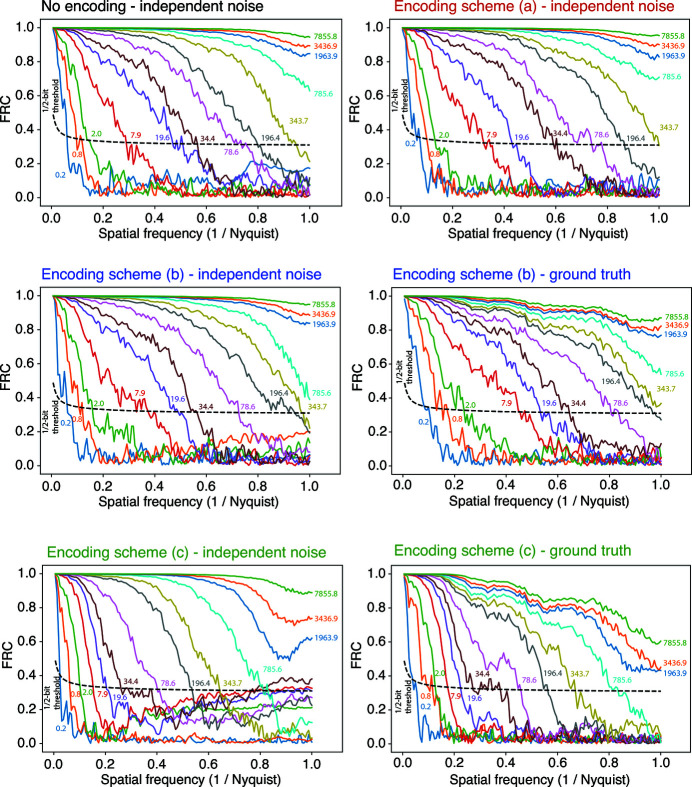
Fourier ring correlation (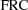) curves for images reconstructed from simulated ptychography datasets with Poisson noise included. For each line color, the associated fluence 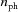 in photons per pixel is indicated. The horizontal axis is shown as a fraction of the normalized spatial frequency 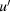 of equation (10). Each curve is labeled with the fluence of averaged incident photons per pixel. Also shown on the plot is the 1/2-bit threshold curve, of which the crossing spatial frequency with the 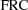 curve is commonly used to define the achieved spatial resolution of the reconstructed image. The dependence of crossing in spatial frequency on fluence is shown in Fig. 8 ▸. These curves are shown for the case of detector intensities recorded as-is (with no encoding) at upper left, and then for the lossy compression schemes (*a*), (*b*), and (*c*) in successive panels. The 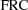 is normally calculated from two separate instances of noisy data (van Heel, 1987 ▸) which is the case labeled ‘independent noise’ for schemes (*a*), (*b*), and (*c*), but we also show the case of comparison of a reconstruction from noisy data compared with the true object in the cases labeled ‘ground truth’ for schemes (*b*) and (*c*). This is done because there is some spurious correlation with a scan grid artifact visible in the rise of the ‘independent noise’ 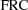 curves at high spatial frequency for schemes (*b*) and (*c*).

**Figure 8:**
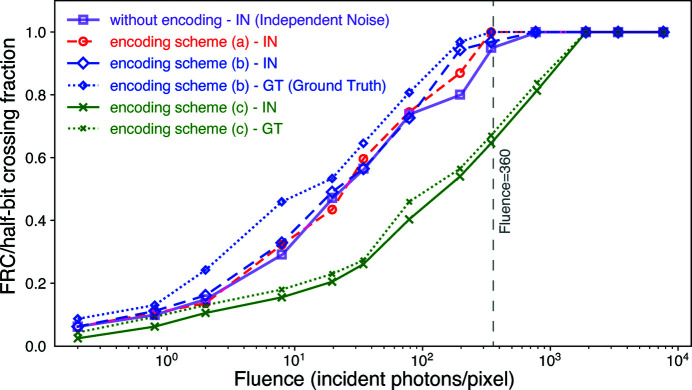
Values of the crossing point between the 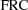 curves of Fig. 7 ▸ and the 1/2-bit threshold are shown here to indicate the achieved spatial resolution of the reconstructed image. The spatial resolution shown on the vertical axis is represented as the fraction of the Nyquist spatial frequency limit, or normalized spatial frequency 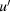 of equation (10). As one approaches the critical fluence of 360 photons pixel^−1^ as estimated using equation (7), the 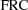 crossing from images obtained from the unencoded data approaches 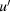 = 1, meaning the achieved resolution is the pixel size of the image. Lossy compression schemes (*a*) and (*b*) produce no decrease in achieved resolution, while scheme (*c*) produces a noticeable decrease in resolution. As in Fig. 7 ▸, the results are shown for the FRC calculated from comparisons between two independent noisy datasets, or one noisy dataset against ground truth.

Another common metric for image evaluation is the Fourier ring correlation (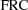), which measures the correlation in phase of two noisy images as a function of spatial resolution (that is, as a function of spatial frequency *u* in the Fourier transform representation *F* of an image) (Saxton & Baumeister, 1982 ▸; van Heel, 1987 ▸). The maximum spatial frequency 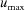 corresponds to the Nyquist limit and is given by
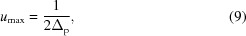
where 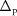 is the pixel size; the normalized spatial frequency 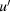 of
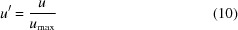
with a range of 0 to 1 is used in Figs. 7 ▸ and 8 ▸. The 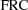 is calculated from *F*_1_ and the complex conjugate 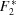 as
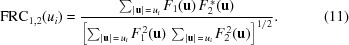
As noted above, an 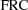-like metric involving one noisy image and the ground truth image was used to measure the performance of the lossy encoding-decoding scheme of equations (2) and (3) as 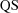 was varied (Wakonig *et al.*, 2020 ▸). Except where noted, we used instead two noisy images from two separately generated Poisson-noise-included datasets, leading to image Fourier transforms 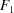 and 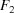 so that one can use equation (11) to obtain the 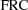 [the 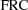 and the 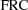-like metric involving the ground truth image give similar trends over changes in fluence (Du *et al.*, 2020 ▸)]. At low spatial frequency *u*, two independent reconstructed images with low fluence will have high correlation due to the same rough outline of the feature existing even for poor-resolution images. Since one needs more fluence to see finer features in coherent X-ray imaging (Huang *et al.*, 2009 ▸; Schropp & Schroer, 2010 ▸; Godard *et al.*, 2012 ▸; Jahn *et al.*, 2017 ▸; Hagemann & Salditt, 2017 ▸; Du *et al.*, 2020 ▸), it is common to use the crossing between the 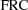 and a 1/2 bit threshold criterion (van Heel & Schatz, 2005 ▸) as a measure of the achieved spatial resolution in an image. For the 1/2 bit threshold, we calculated the FRC between two full images instead of two half images as described by van Heel & Schatz, (2005 ▸). The derivation of the 1/2 bit threshold formula used in this work is included in the supporting information. We show here in Fig. 8 ▸ this 1/2 bit 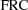 crossing point as a function of fluence for images reconstructed from the non-encoded data, and also from images reconstructed from data with lossy compression schemes (*a*), (*b*), and (*c*). As one approaches the critical fluence of 360 photons pixel^−1^ estimated from equation (7), the 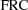 crossing point nears 1 (meaning the image is reconstructed at the resolution of the pixel size) for the unencoded data, and also for lossy compression schemes (*a*) and (*b*). Again, one sees essentially no change in a metric of reconstructed image quality with lossy encoding schemes (*a*) and (*b*), but as with the 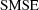 error one does see a significant degradation when using lossy encoding scheme (*c*).

## 6. Conclusion   

Compression of ptychographic datasets is important not only for decreasing the amount of storage space they require on disk (Loetgering *et al.*, 2017 ▸) or in GPU memory (Wakonig *et al.*, 2020 ▸) but it can also be used to decrease the bandwidth required for streaming high frame rate data from a hybrid pixel array detector (HPAD) on a limited-bandwidth communication channel. Compression can be provided in a FPGA attached to an ASIC, but this comes at the cost of higher power consumption (Amara *et al.*, 2006 ▸) and furthermore it does not offer simplification in data transfer wiring within the ASIC as noted in Section 3. We have therefore described lossy compression schemes that involve simple bit shifts and additions [equations (4) and (5)], so that they can be implemented on already-digitized data on a per-pixel basis even given the limited circuitry area available per pixel on a HPAD application-specific integrated circuit (ASIC). By keeping the compression loss to a value below one standard deviation of the Poisson distribution, we achieved a reduction of the data size to 64% with scheme (*a*) and 57% with scheme (*b*). With both of these schemes, the images reconstructed from simulated ptychographic data over a wide range of photon fluences show no degradation in reconstructed image quality as measured by a standard mean squared error (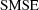), and no degradation in the spatial resolution as estimated using Fourier ring correlation (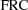).

This provides a potential pathway for increasing the usable frame rate of HPADs as will be required for scaling ptychography up to imaging larger objects, and thus exploiting the high penetrating power of X rays.

## Supplementary Material

Supplementary material. DOI: 10.1107/S1600577520013326/gy5014sup1.pdf

## Figures and Tables

**Table 1:** Lossy encoding scheme for representing a photon count 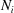 in an encoded value 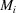 using equation (4), and obtaining the decoded value 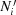 using equation (5) The values for 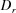 and 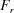 used here are for scheme (*a*) shown in Fig. 1 ▸. The error 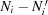 is always less than the standard deviation 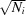 of the Poisson distribution, as shown in the final column. This lossy encoding scheme is particularly easy to implement for integer operations in per-pixel ASIC hardware.

Range	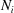	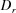	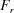	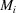	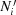	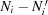	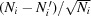
1	0	1	0	0	0	0	0.00
	15			15	15	0	0.00
2	16	4	16	20	16	0	0.00
	63			31	60	3	0.38
3	64	8	32	40	64	0	0.00
	255			63	248	7	0.44
4	256	16	64	80	256	0	0.00
	1023			127	1008	15	0.47
5	1024	32	128	160	1024	0	0.00
	4095			255	4064	31	0.48
6	4096	64	256	320	4096	0	0.00
	16383			511	16320	63	0.49
