# Supplementary material for: Fast digital lossy compression for X-ray ptychographic data
Source: J Synchrotron Radiat. 2021 Jan 1;28(Pt 1):292–300. doi: 10.1107/S1600577520013326 (PMC7842218; doi:10.1107/S1600577520013326)
Supplement: Supplementary file 1 [file s-28-00292-sup1.pdf]

Supplementary Material: Fast Digital Lossy Compression for X-ray Ptychographic Data

The crossing between Fourier ring correlation (FRC) and 1/2 bit threshold is a widely used standard to determine the achieved resolution of the reconstructed image in ptychography. Here we derive the formula of 1/2 bit threshold when the FRC is calculated using 2 full images with independent noise.

Given the required SNR, the corresponding Fourier correlation (which in the 2D case is a Fourier ring correlation or FRC rather than a Fourier shell correlation) can be calculated using Eq. 13 of [1], which we rewrite here as

$$\mathrm{FRC}(r_i) = \frac{\mathrm{SNR}(r_i) + 2/\sqrt{n(r_i)}\sqrt{\mathrm{SNR}(r_i)} + 1/\sqrt{n(r_i)}}{\mathrm{SNR}(r_i) + 2/\sqrt{n(r_i)}\sqrt{\mathrm{SNR}(r_i)} + 1} \tag{1}$$

where $n(r_i)$ is the number of voxels in the Fourier shell, r_i. The required SNR of the 1/2-bit threshold can be calculated using

$$\frac{1}{2}\,\mathrm{bit} = \log_2(1 + \mathrm{SNR}) = \log_2(1 + 0.4142) \tag{2}$$

from which we obtained the requirement of SNR = 0.4142 in the full image.

In the case of calculating the FRC between 2 half images, the required SNR is in each of the half images is also half (0.4142/2 = 0.2071) because the total SNR is twice the SNR value of each of the half images. The 1/2-bit threshold then can be calculated by plugging $\mathrm{SNR}(r_i) = 0.2071$ into Eq. (1) to get Eq. 17 of [1], which we rewrite here as

$$T_{1/2-\mathrm{bit}} = \frac{0.2071 + 1.9102/\sqrt{n_{r_i}}}{1.2071 + 1.9102/\sqrt{n_{r_i}}}. \tag{3}$$

In our work, we calculated the FRC between 2 full images. Therefore, we plugged the value SNR = 0.4142 directly into Eq. (1), which gives

$$T_{1/2\text{-bit}} = \frac{0.4142 + 2.2872/\sqrt{n_{r_i}}}{1.4142 + 1.2872/\sqrt{n_{r_i}}}. \tag{4}$$

References

[1] Marin van Heel and Michael Schatz. Fourier shell correlation threshold criteria. Journal of Structural Biology, 151(3):250–262, September 2005.
